# Papillary carcinoma arising in a thyroglossal duct cyst with associated microcarcinoma of the thyroid and without cervical lymph node metastasis: a case report

**DOI:** 10.1186/1752-1947-2-42

**Published:** 2008-02-08

**Authors:** Tolga Kandogan, Nazif Erkan, Enver Vardar

**Affiliations:** 1 Department of Otolaryngology, Izmir Training and Research Hospital, Bozyaka İzmir 35290 Turkey; 2 Department of Surgery, Izmir Training and Research Hospital, Bozyaka İzmir 35290 Turkey; 3 Department of Pathology, Izmir Training and Research Hospital, Bozyaka İzmir 35290 Turkey

## Abstract

**Introduction:**

This is a case report of a 44-year-old woman with papillary carcinoma of a thyroglossal duct cyst.

**Case presentation:**

A 44 year-old woman presented to the otolaryngology outpatient clinic with an asymptomatic anterior midline neck mass. A cervical ultrasound showed a lesion which appeared to be a thyroglossal duct cyst and surgical resection using Sistrunk's procedure was performed. The histopathologic diagnosis showed papillary carcinoma evolving from a thyroglossal duct cyst, confined to the thyroglossal cyst, with a tumor diameter of 2 cm. The patient then underwent total thyroidectomy and bilateral neck dissection. The final pathology reported an 8 mm papillary cancer in the left lobe of the thyroid without any metastasis to the cervical lymph nodes. The patient was treated with radioactive iodide and thyroid suppresion therapy was given as adjuvant treatment. The patient has been following for two years without any metastasis.

**Conclusion:**

Malignancy within a thyroglossal duct cyst is very rare but should be considered in the differential diagnosis of a midline neck mass.

## Introduction

As the thyroid gland descends from the foramen cecum to its location at the point below the thyroid cartilage, it leaves behind an epithelial trace known as the thyroglossal tract. The tract disappears during the 5th-10th gestational week. Incomplete atrophy of the thyroglossal tract, or retained epithelial cysts, creates the basis for the origin of a thyroglossal duct cyst (TGDC). A thyroglossal remnant can be a cyst, a tract or duct, a fistula, or an ectopic thyroid within a cyst or duct [1].

A TGDC is the most common anomaly in the development of the thyroid gland [2]. 70% are diagnosed in childhood and 7% are diagnosed in adulthood [3]. Only 1% of thyroid carcinomas arise from a TGDC [4].

In this report, we present a female adult with a papillary carcinoma of the TGDC.

## Case presentation

A 44-year-old woman presented to the otolaryngology outpatient clinic with an asymptomatic anterior midline neck mass. The tumor had developed over 6 months. Physical examination revealed a 2 × 2 cm mass on the anterior part of the neck between the thyroid cartilage and hyoid. She was in good health otherwise and her past medical history was unremarkable.

**Figure 1:**
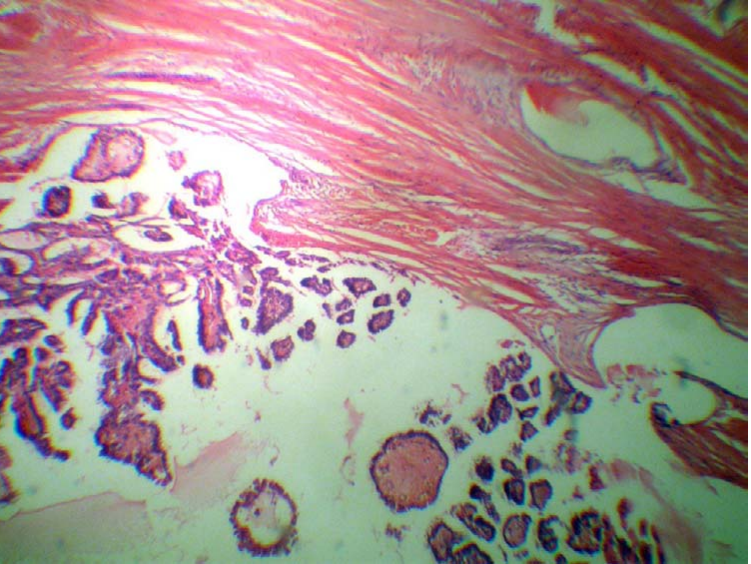
Papillary carcinoma evolving from a TGDC, confined to the thyroglossal cyst. (H&E).

**Figure 2:**
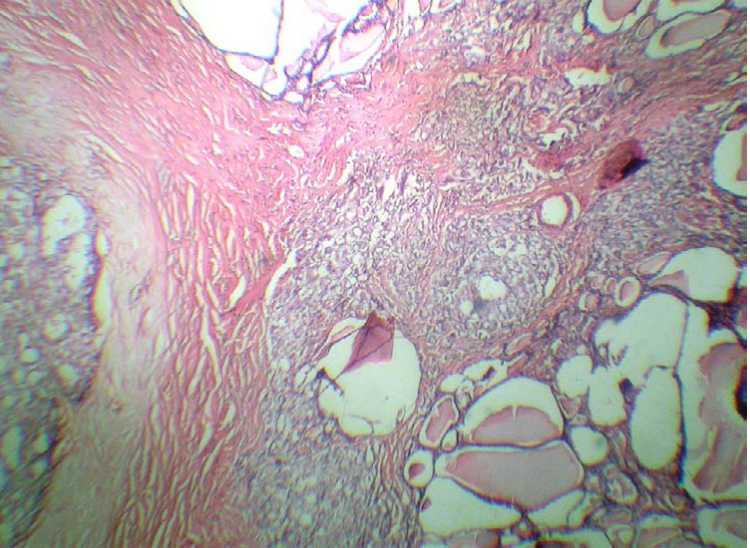
Papillary cancer in the left lobe of the thyroid. (H&E).

A cervical ultrasound showed a lesion which appeared to be a TGDC and surgical resection by means of Sistrunk's procedure was performed. The histopathologic diagnosis was a papillary carcinoma evolving from a TGDC, confined to the thyroglossal cyst with a tumor diameter of 2 cm (Figure 1). The patient was referred to the surgery department for further investigation. Thyroid scintigraphy, ultrasound and cervical CT scans were performed. The thyroid scintigraphy was normal. The cervical ultrasound showed multiple cervical lymph nodes which were of different sizes ranging from 8 mm to 17 mm. A cervical CT revealed bilateral cervical lymph nodes less than 2 cm in diameter. The patient's thyroid function tests were within normal limits. Total thyroidectomy and bilateral neck dissection were performed. The final postoperative pathology reported an 8 mm papillary cancer in the left lobe of the thyroid without any metastasis to the cervical lymph nodes (Figure 2). The patient was treated with radioactive iodide and thyroid suppresion therapy was given as an adjuvant treatment. The patient has been following without any metastasis for two years.

## Discussion

A mass in the neck is a common clinical finding and differential diagnosis may be extremely broad. Although most masses are due to benign processes, malignant diseases must not be overlooked. Therefore, it is important to develop a systematic approach for the diagnosis and management of neck masses.

Benign thyroglossal duct cysts usually present as aysmptomatic, soft, firm, or hard masses in the midline of the anterior neck, and are nontender and generally movable. Malignant thyroglossal duct cysts present in the same manner. Carcinoma should be suspected in any thyroglossal duct cyst that is hard, fixed and irregular or which has undergone recent change. A history of irradiation of the head and neck or mediastinum during childhood or adolescence sholud also arouse suspicion of carcinoma [1].

Malignant tumors developing from the thyroglossal duct have two origins: thyrogenic carcinoma arising from thyroembrionic remnants in the duct or a cyst, and squamous cell carcinoma arising from metaplastic columnar cells that line the duct [1]. More then 200 cases of thyroglossal duct carcinomas have been reported in which papillary carcinoma accounts for 80% of cases, with the rest being squamous cell carcinoma [5-7]. Only one case with both concomitant histologic findings has been reported [8].

Excluding medullary carcinoma, which arises from parafollicular cells embryologically unrelated to the thyroid, all forms of primary thyroid carcinoma can arise in the thyroglossal duct [1].

The main difficulty encountered with a cancer evolving from a thyroglossal duct cyst is that the diagnosis is usually made during surgery or from definitive pathological samples. Because the frequency of cancer of the thyroglossal duct cyst is very low, the clinician often does not consider an oncologic diagnosis. A second difficulty lies in terms of what approach should be taken during and after surgery when dealing with a preoperatively diagnosed thyroglossal cyst; that is, how extensive should the surgery be and what type of adjuvant therapy should be used [9]? To be able to respond to these two issues, the procedure used for thyroglossal cyst surgery must be standarized.

When a thyroglossal duct cyst has been excised using Sistrunk's procedure and when the definitive hystological analysis reports malignancy, the thyroid gland must be studied with radiological and scintigraphic examinations and the extension of surgery must be handled according to the criteria established for differentiated thyroid cancer [9]. In our case, we made a radical surgical method with total tyroidectomy and bilateral neck dissection due to findings on cervical CT.

The common surgical procedure used for a thyroglossal duct cyst is Sistrunk's procedure, consisting of excision of the thyroglossal duct cyst, the central portion of the body of the hyoid bone, and a core of tissue around the thyroglossal tract to open into the oral cavity at the foramen cecum[1]. In case of malignancy, additional steps should consist of thyroidectomy, radioactive iodine and thyroid supression, as is the case for differentiated thyroid cancers.

## Conclusion

Malignancy within a thyroglossal duct cyst is very rare but should be included in the differential diagnosis of a neck mass. This condition is rarely diagnosed preoperatively. Once diagnosed, therapy includes surgery, radioactive iodine and thyroid supression, as is the case for differentiated thyroid cancers.

## Competing interests

The author(s) declare that they have no competing interests.

## Authors' contributions

TK, NE and EV drafted the manuscript and designed the case report. All authors read and approved the final manuscript.

## Consent

Written informed patient consent was obtained for publication.
